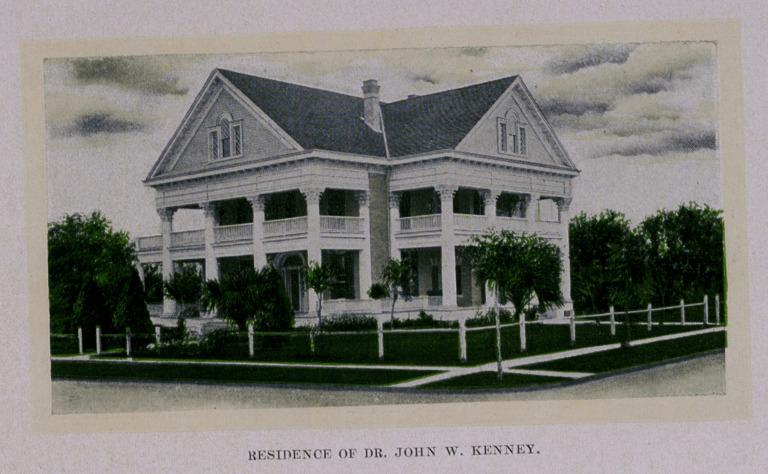# Gastroenterostomy for Drunkenness

**Published:** 1910-09

**Authors:** 


### EDITORIAL DEPARTMENT.

### GASTROENTEROSTOMY FOR DRUNKENNESS.

### DR. J. W. KENNEY AND HIS SANATORIUM AND TRAINING SCHOOL FOR NURSES.

A grave surgical operation for the cure of chronic alcoholism and eradication of the craving is rather a startling proposition; yet it has been done with success in a number of cases and is being done by Drs. J. W. and Nat. Kenney at their sanatorium in San Antonio.

Gastroenterostomy was introduced for the relief of stricture or other obstruction at the pylorus, and I did not suppose that it is ever resorted to or is justifiable for anything else, but Dr. Kenney conceived the idea that if an opening between the jejunum and the lowest portion of the great curvature of the stomach were made it would serve something like drainage does in wounds, and give the ulcerated and inflamed mucous walls of the stomach a rest, and chance to heal. He had a great curiosity to try it. He had an idea that surgical treatment might offer a chance of cure,—of the gastritis at least; but, I do not suppose that he dreamed that it would cure the drink craving (dipsomania, a neurosis, or neuro-psychosis). There are more things in a doctor's heaven and earth than were ever dreamed of in Horatio's philosophy. The opportunity presented itself, and Dr. Kenney seized it. A hopeless drunkard—a pauper—applied to Dr. Kenney and agreed to submit to the treatment. It was successful, the man's health was restored and he was *cured of the craving!*

Since the results of the case, a perfect cure, Dr. Kenney has operated on sixteen other drunkards with complete cure in fifteen (sixteen in all). Of the seventeen cases operated on one died, Dr. Kenney states; not from the effects of the operation, but of angina pectoris, to which the patient was subject.

He operated very recently on Mr. Jas. Thornton, a well known young man of Austin, for the relief of EPILEPTIFORM CONVULSIONS, believed to be epilepsy, and treated for such by the ablest physicians North, South, East and West without benefit. He found hour-glass contraction of the stomach—stricture, and operated, establishing a gastro-jejunal opening, and is awaiting results before reporting the case. If he has found the cause of Mr. Thornton's trouble, a reflex from a diseased stomach—and removed it, it will throw some light upon the nature of epilepsy and show that it is not always a brain disease.

However, this is a little premature. Dr. Kenney will contribute to the TEXAS MEDICAL JOURNAL for October a clinical report of the seventeen cases operated on for drunkenness, illustrated by original drawings; and will report the Thornton case as soon as results are known.*

The remarkable discovery by Dr. Kenney that a gastroenterostomy will cure drunkenness and has done so at his hands has given the well known sanatorium quite a local notoriety, and has aroused much interest in the professional ranks. San Antonio may become the Mecca to which the incurable drunkards will journey, and surgeons to see the work and its results. The writer went to San Antonio for the purpose. He is promised opportunity to see the next case operated on. While there he personally inspected this splendid institution—already widely known, and was so pleased that he devotes this space to it. It is a credit to San Antonio and to the State, and the JOURNAL is proud of the talented young physicians who have made it famous.

The beautiful buildings herewith pictured are situated on Tobin's Hill, the swell, aristocratic residence district of the city. There is an air of home-like comfort, quiet, restfulness and seclusion,—privacy,—about the sanatorium, while the palatial residence of the Doctor crowns the scene like a diadem. Dr. Kenney is one of the larget real estate owners in San Antonio, and has done much to advance the prosperity and development of the splendid semi-tropical metropolis. I went through the sanatorium and inspected every department, and it is above criticism in every detail. It is needless to say that the equipment in every department is complete and every appliance for treatment, medical and surgical, is there, and of the latest,—aseptic operating room, the latest instruments and fixtures, electrical, including the X-Ray and "artificial sunshine" baths (electric lights), medicated and other baths for hydro-therapy, etc. Everything was scrupulously clean and restful. It looked like it would almost be a luxury to lie in one of the snowy beds—between the large open windows through which the sweet south wind wafts the perfume of thousands of roses and the blossom of the woodbine and the jasamine, the tube-

---

*Mr. Thornton is not a drinker.

COUNTRY PLACE—DR. KENNEY'S SANATORIUM.

rose and the lily, and to have cool drinks and tempting "nourishment" served by unusually pretty, intelligent and attractive nurses, in spotless garb and dainty muslin caps,—oh, well, and these nurses—all graduates of well known training schools—are veterans. They are assisting the Drs. Kenney in training others --younger ones—for home consumption, I may say; for in connection with the Sanatorium Drs. Kenney have a

TRAINING SCHOOL FOR NURSES.—Chartered by the State and the novitiates go through a three-years' course of instruction by lectures—didactic and clinical, laboratory courses, microscope, chemical, and especially are they taught the fine art of dietetics, preparation of "nourishment" for the sick. They receive a diploma, and are immediately "put on the wards." There are twenty of these white-capped ministers of mercy on duty there at this time.

The country place (see cut) is seven miles from the city, and is equipped and reserved for nervous cases—border-land cases—"mildly insane," who need and must have quiet. No contagious or infectious cases are taken at either place, the main institution being mostly devoted to surgical cases.

Dr. Kenney will gladly send an illustrated booklet on request, containing full details as to terms, etc. Drop him a postal.

P. S.—Since the above was put in type Mr. Thornton was killed in an automobile accident.

## Figures and Tables

**Figure f1:**
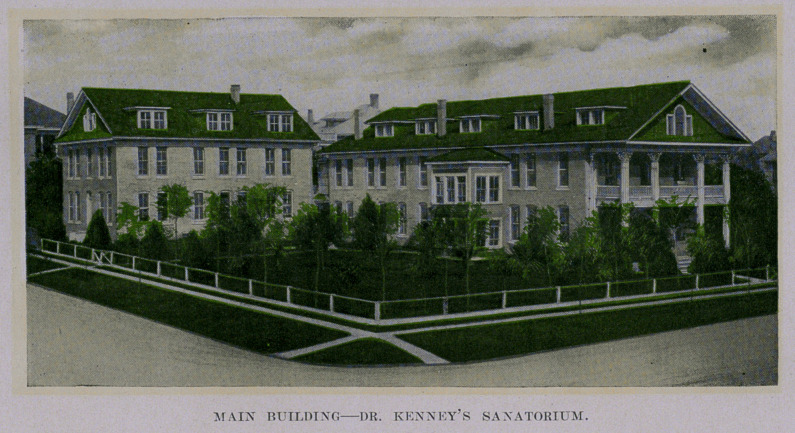


**Figure f2:**
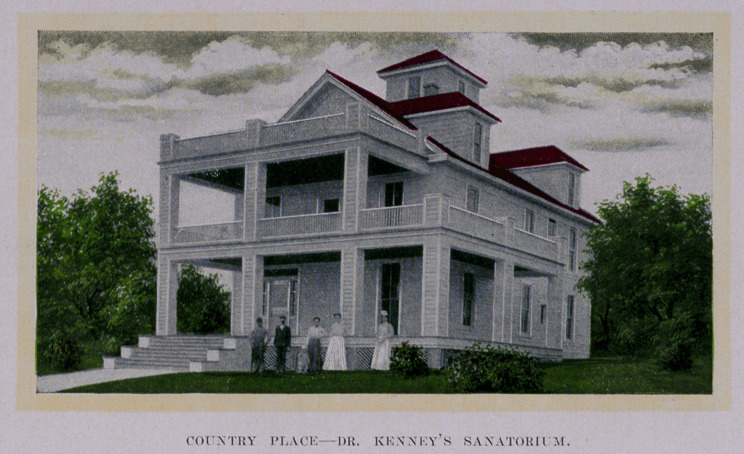


**Figure f3:**